# A critical review of the use of R^2^ in risk equalization research

**DOI:** 10.1007/s10198-024-01709-8

**Published:** 2024-08-09

**Authors:** Wynand P. M. M. van de Ven, Richard C. van Kleef

**Affiliations:** Erasmus University Rotterdam / Erasmus Centre for Health Economics Rotterdam (EsCHER), Rotterdam, The Netherlands

**Keywords:** R^2^, Risk equalization, Health insurance, Mean absolute prediction error (MAPE), Cumming’s prediction measure (CPM), Payment system fit (PSF), C10, G22, I11, I13

## Abstract

Nearly all empirical studies that estimate the coefficients of a risk equalization formula present the value of the statistical measure R^2^. The R^2^-value is often (implicitly) interpreted as a measure of the extent to which the risk equalization payments remove the regulation-induced predictable profits and losses on the insured, with a higher R^2^-value indicating a better performance. In many cases, however, we do not know whether a model with R^2^ = 0.30 reduces the predictable profits and losses more than a model with R^2^ = 0.20. In this paper we argue that in the context of risk equalization R^2^ is hard to interpret as a measure of selection incentives, can lead to wrong and misleading conclusions when used as a measure of selection incentives, and is therefore not useful for measuring selection incentives. The same is true for related statistical measures such as the Mean Absolute Prediction Error (MAPE), Cumming’s Prediction Measure (CPM) and the Payment System Fit (PSF). There are some exceptions where the R^2^ can be useful. Our recommendation is to either present the R^2^ with a clear, valid, and relevant interpretation or not to present the R^2^. The same holds for the related statistical measures MAPE, CPM and PSF.

## Introduction

Regulated health insurance markets are often characterized by premium regulation and open enrolment. Although these regulations can help improve the affordability and accessibility of health insurance coverage for high-risk people, they also create predictable profits and losses on insured, and therefore provide insurers with incentives for risk selection. To prevent the adverse effects of risk selection, a risk equalization scheme can be implemented. That is a system of risk adjusted equalization payments to and from the insurers that compensate insurers for differences in individuals’ expected healthcare expenses that are not allowed to be explicitly reflected in premium differences. The goal of risk equalization is to eliminate the regulation-induced incentives for risk selection^1^ by compensating insurers for predictable profits and losses [2].^2^ Therefore, an important aspect of the evaluation of risk equalization formulas is the extent to which the risk equalization payments remove the regulation-induced *predictable* profits and losses on the insured.

With *predictable* profits and losses we mean the profits and losses that, given the premium regulation, can be predicted by consumers and/or insurers prior to the contract period. That is, there is an information asymmetry in the sense that consumers and/or insurers have more predictive information about future healthcare expenses than the information that is used for calculating the equalization payments. Insurers have rich panel data that they can use to make better predictions of the future individual healthcare expenses than the predictions based on imperfect risk equalization. Insurers can use their information surplus for risk selection, which can have negative effects in terms of quality of care, efficiency, and affordability [e.g., 3]. Insurers can learn about predictable profits and losses in subtle ways. For example, any correlation between consumers’ preferences regarding health insurance and their health risk can reveal a predictable profit or loss. When insurers know these correlations (e.g., based on revealed preferences in a prior period), they can exploit the associated predictable profits and losses via the design of health insurance products. In addition, insurers can base their selection strategy on the information that is increasingly presented in the literature about which groups of people generate predictable profits or losses, given a certain equalization formula [e.g., 3 and 5–11]. And, finally, insurers don’t need to know which specific individuals are (un)profitable, it is sufficient for them to know that *there are* (un)profitable individuals. They can then use general selection tools, e.g., different levels of deductibles, the copayment structure and benefits design. Consumers will then use their information surplus for choosing the most appropriate health insurance product (adverse selection). Of course, to prevent selection the risk equalization needs only to compensate for predictable profits and losses as far as insurers and/or consumers can exploit them by selection actions. But it is hard to think of any predictable profits or losses that cannot be exploited by selection actions.^3^

The coefficients of the risk equalization formula are typically estimated by means of the Ordinary Least Squares (OLS) regression method with individual healthcare expenses as dependent variable and a set of risk adjusters as explanatory variables. OLS finds the set of coefficients that minimizes the “sum of squared residuals”, given the set of risk adjusters. The statistical software used to run OLS regressions typically also presents the so-called R^2^.^4^ The R^2^-value indicates the proportion of the total variance in individual expenditures that is explained by the linear influence of the set of risk adjusters, and ranges between zero and one.^5^ In empirical evaluations of risk equalization models, researchers routinely present the R^2^-value, but often without an explicit interpretation. More specifically, researchers and policymakers often implicitly use the R^2^ as a measure to evaluate the extent to which a risk equalization model reduces *predictable* profits and losses, with a value closer to one indicating better performance.

The goal of this paper^6^ is to critically review the use of R^2^ in conventional risk equalization research, i.e., research in which first the weights for a given risk equalization (or prediction) model are estimated and then second the risk equalization model is evaluated based on certain criteria that reflect the regulator’s objective. In Sect. "R2 is hard to interpret" we discuss why the R^2^ is hard to interpret as a measure of selection incentives and can lead to wrong and misleading conclusions regarding the extent to which a risk equalization model reduces predictable profits and losses. In many cases, we do not know whether a risk equalization model with R^2^ = 0.30 reduces the predictable profits and losses more than a model with R^2^ = 0.20. In Sect. "Related measures-of-fit: similar problems" we argue that part of our critique on using the R^2^ as a performance measure that indicates the extent to which a risk equalization model reduces *predictable* profits and losses, also holds for other statistical metrics such as the Mean Absolute Prediction Error (MAPE), Cumming’s Prediction Measure (CPM), and the Payment System Fit (PSF). While the R^2^ is inappropriate for indicating the extent to which a risk equalization model mitigates predictable profits and losses, it can be useful for other purposes, which will be discussed in Sect. "Applications in which R2 can be useful". Sect. "Conclusion and discussion" summarizes and discusses the conclusions.

## R^2^ is hard to interpret

Nearly all empirical studies that quantitatively evaluate the performance of risk equalization models present the statistical R^2^-value [12]. The R^2^-value is often (implicitly) interpreted as a measure of the extent to which the risk equalization payments remove the regulation-induced ‘predictable profits and losses on the insured’ (i.e., the incentives for selection), with a higher R^2^-value indicating a better performance. For three reasons, however, the R^2^-value is hard to interpret as a measure of selection incentives. First, the maximum R^2^-value is typically unknown. Consequently, the R^2^-value does not indicate to what extent predictable profits and losses remain after risk equalization. Second, the R^2^-value is nonlinearly related to the predictable profits and losses. As a result, a change in R^2^-value due to the inclusion of a new risk adjuster does not indicate the extent to which that risk adjuster contributes to the reduction of predictable profits and losses. Third, a high R^2^-value does not necessarily imply smaller predictable profits and losses than a low R^2^-value. Consequently, comparing alternative equalization models solely on the basis of the R^2^-value can lead to misleading conclusions about which model is to be preferred. Below, we explain these three problems in more detail.

### No benchmark because the maximum R^2^-value is typically unknown

The R^2^ indicates the proportion of the *total* variance in individual healthcare expenses that can be predicted by the risk adjusters. A major problem with the interpretation of the R^2^-value is that a substantial part of the variance in individual healthcare expenses is *not predictable,* due to e.g., accidents and the unforeseen onset of new diseases, and thus cannot be anticipated on by insurers and consumers. Therefore, when assessing the performance of a risk equalization model we are more interested in the proportion of the *maximum predictable* variance that is predicted by the risk adjusters than in the proportion of the *total* variance that is predicted by the risk adjusters (= R^2^-value). However, in general we do not know the maximum R^2^-value for a specific setting. This lack of a benchmark makes R^2^ hard to interpret and an inappropriate measure of the extent to which the risk equalization payments remove the regulation-induced *predictable* profits and losses*.* More specifically, the R^2^-value does not indicate to what extent predictable profits and losses remain after risk equalization.

Some studies have dealt with the question what the maximum R^2^ is that can be achieved by a set of prospective^7^ risk adjusters in a specific setting.^8^ These studies are based on different assumptions about the anatomy of the variance of the actual expenses. Newhouse et al. [14]) and Newhouse [1] assumed that the variance of the actual expenses can be divided into three components^9^:

1. The first component is a *fixed* effect, i.e., the constant above or below average expenses of a person over the last years. For the analyzed data set this component has been estimated to be at most 15–20% of the total variance.
2. The second component is a predictable, but *time-varying* effect, i.e., the above or below average expenses of a person in the next period but that would not persist in later periods. This component is estimated to be another 3–5% of total variance.
3. The third component is the *random* component, i.e., the unpredictable variance.

Newhouse [1] concludes that with risk factors based on observed individual utilization/expenses in prior periods one can explain around 20–25% of the variance of annual actual expenses. However, this percentage is a lower bound of the maximum predictable variance because there may be additional predictive information that is not reflected in past utilization/expenses (e.g., giving birth in the next period). Therefore, consumers or insurers could potentially predict more than the 20–25%, but how much more is unknown.

Van Vliet [16] compared four different error component models and concluded that for the analyzed data set ‘no more than 20% of annual costs variations are predictable at the individual level’.

One should be careful in using the maximum R^2^ estimated in one setting as a benchmark in other settings because the maximum R^2^ is context specific and may depend on the following determinants [3, p.790–793]:

- *The type of services under analysis.*
  For example, outpatient care and pharmaceutical care are typically more predictable than inpatient care, and long-term care is typically more predictable than acute care [3, p.790]. This implies that for one set of services the maximum R^2^ can be lower/higher than for another, ceteris paribus.
- *The (sub)population under analysis.*
  Kronick et al. [17] concluded that expenditures are much more predictable among persons with chronic conditions than for other populations. They hypothesize that among persons with chronic conditions (disabilities or diseases) a much greater portion of resource utilization results from chronic problems and their complications that persist from year to year, and a smaller portion from acute episodes that lead to short-term spikes in resource use but are not followed by long-term needs. The same holds for age, with elderly people having on average more chronic diseases than young people. Newhouse et al. [14 and 18] and Van Vliet [16] found empirical evidence that differences in health expenditures for older individuals are more predictable than those for young people. This implies that for one population the maximum R^2^ can be lower/higher than for another, ceteris paribus.
- *The price of healthcare services.*
  A change of the price of healthcare services may influence the R^2^-value. For example, Van Kleef et al. [19, p. 414–5] show that a substantial reduction of the price for kidney dialysis (a treatment that also formed the basis for a risk adjuster in the risk equalization formula), ceteris paribus resulted in a substantial decrease in the R^2^-value (from about 0.25 to 0.20). This implies that for one set of healthcare prices the maximum R^2^ can be lower/higher than for another, ceteris paribus.
- *The level of effective medical technology.*
  Van de Ven and Ellis [3, p. 791] hypothesize a positive relation between R^2^ and the level of medical technology. An increase of the level of effective medical technologies may keep alive at-risk patients who otherwise would have died, and thereby increases the proportion of chronically ill persons. Their healthcare expenses are better predictable than those of healthy people without chronic problems (see above). This implies that for one level of medical technology the maximum R^2^ can be lower/higher than for another, ceteris paribus.
- *The length of the period being predicted.*
  By using a longer period for predicting healthcare expenses (e.g., a year rather than a month) some of the random variation is averaged out, which—under the assumption that the systematic variation largely remains unchanged—increases the R^2^. This implies that for one length of period the maximum R^2^ can be lower/higher than for another, ceteris paribus.

Because of these many determinants, the maximum R^2^-value can substantially differ across settings and over time. For example, the above-mentioned results about the maximum R^2^-values presented by Newhouse [1] and Van Vliet [16] are based on data sets from the 1970s. These estimated maximum R^2^-values seem no longer relevant, as recent studies that estimate a prospective risk equalization model based on data sets from forty years later find R^2^-values somewhere in the range of 0.30–0.35. For example, Van Kleef et al. [20] found an R^2^ of 0.30, which is clearly below the maximum R^2^, because they also found that the risk equalization model studied in their paper undercompensates insurers for chronically ill people.

So, due to a lack of a benchmark one should be careful in using the R^2^ as a measure to indicate the extent to which a risk equalization model reduces the regulation-induced predictable profits and losses. Ideally, to have a benchmark researchers should estimate the maximum R^2^ on the same (longitudinal) data base that is used for estimating the coefficients of the risk equalization model. However, although the formulas how to do so are known, in practice this is hardly done,^10^ implying that the R^2^-value does not indicate to what extent *predictable* profits and losses remain after risk equalization.

### R^2^ is nonlinearly related to the predictable profits and losses

Even if we knew the maximum R^2^, the R^2^-value would still be hard to interpret. An x-percent decrease of the difference between R^2^ and the maximum R^2^, as a result of an improvement of the equalization formula, cannot be interpreted as an x-percent decrease of the predictable profits and losses, because the relation between R^2^ and the predictable profits and losses is nonlinear. From the point of view of the regulator, this nonlinearity is in exactly the wrong direction because the last percentage point of variance explained may result in a larger reduction of the predictable profits and losses than the earlier changes [1, 7, 14]. For example, Van Barneveld et al. [7, p. 134] found that the first five percentage points increase in the R^2^ lead to a reduction of the mean absolute predicted result (= profit/loss) of 360 Dutch guilders, whereas the last five percentage points increase in the R^2^ lead to a reduction of 720 Dutch guilders. Newhouse et al. [14] found similar results.^11^

The quadratic weighing of residuals (i.e., the insurers’ actual profits and losses) in the R^2^ results in a nonlinear relation between R^2^ and the predicted profits/losses, and can lead to misleading conclusions in the case of *small* predictable profits and losses. Although large residuals may be more problematic than small residuals, it is not obvious that quadratic weighting is better for the evaluation of the regulation-induced profits and losses than alternative forms of weighting. According to Layton et al. [21] the argument in favor of squaring the residuals, such as in R^2^, has a sound basis in welfare economics, where it is normally assumed that the welfare loss of a distortionary incentive is proportional to the square of the distortionary incentive. Although this squaring-argument may hold in the familiar context of a tax [22], it is not clear that it is a valid argument for squaring the residuals as is done in R^2^ in the context of risk equalization. First, the distortionary incentives are the (by consumers and insurers) *predictable* profits and losses and not the *actual* profits and losses (i.e., the residuals), which are much larger and have more extreme outliers (that weigh heavily in the squaring) than the *predictable* profits and losses. The R^2^ is a function of the squared *actual* profits/losses and not of the *predictable* profits/losses. The R^2^ attaches enormous weight to large outliers: the one person in a sample with an actual loss of a million euros will add as much to the variance as 1,000,000 people with an actual loss of 1000 euro [3, p. 810]. Second, in the case of selection incentives one could hypothesize that above a certain level of the distortionary incentive the welfare loss looks more like a flat curve than a quadratic function of the distortionary incentive. The higher the undercompensations and the more stable the undercompensations are over time, the more insurers may respond to the distortionary incentives. However, it could be hypothesized that at a certain level of undercompensation U the insurers have achieved their ‘maximum selection actions’, i.e., they do their utmost best to get rid of unprofitable patients and there are no additional cost-effective selection actions. This would imply that with undercompensations larger than U the welfare loss resulting from risk selection is a flat curve. So, it is not obvious that quadratic weighting is better than alternative forms of weighting.^12^

The quadratic weighing of residuals can lead to misleading conclusions in the case of *small* predictable profits and losses. For example, Van Kleef [23] present results that the addition of socio-economic characteristics to a relatively good performing risk equalization model increases the R^2^ from 0.3438 to 0.3440. The elimination of the relatively small predictable profits and losses on the different socioeconomic groups results in only a seemingly negligible increase of the R^2^. If a regulator would base his decision *solely* on the R^2^-value rounded to three decimal places (0.344) one might conclude not to include these risk adjusters in the risk equalization model. However, a regulator might find the addition of these risk adjusters to be relevant as they eliminate the incentives for risk selection against socio-economic groups and thereby eliminate the negative welfare effects of that type of risk selection. This example illustrates that evaluating (potential) risk adjusters solely on the basis of R^2^ can lead to misleading conclusions.

### A high R^2^ does not necessarily imply a better performance than a low R^2^

According to Layton et al. [24, p. 141–142] it is intuitive that a higher R^2^ should improve the performance of a payment system with respect to selection problems. This reflects the general perception that a payment system with a high R^2^ reduces the predictable profits and losses more than a payment system with a low R^2^. However, this general perception is not necessarily correct. Below we give some counterexamples.

A first example is the comparison of the R^2^-values for risk equalization models that explain different types of healthcare expenses or expenses in different settings. The R^2^-values also depend, just as the maximum R^2^ (see above), on the type of healthcare services, the subpopulation, the price of healthcare services, and medical technology [3, p.790–793]. So, the R^2^ may strongly depend on e.g., whether prescription drugs are included (as e.g., in the Netherlands) or not included (as in Medicare Advantage), whether the health expenses relate to the whole population (as e.g., in the Netherlands) or only to elderly (as in Medicare Advantage), or on the extent to which long term care is included. So, when comparing for, example, a model that predicts healthcare expenses including prescription drugs for an elderly unhealthy population with a model that predicts healthcare expenses not including prescription drugs for a young healthy population, the first model may have a higher R^2^ than the second model. However, that does not necessarily mean that the first model reduces the selection incentives more than the second model. For similar reasons a higher R^2^-value in setting A than in setting B does not necessarily mean that selection incentives are smaller in A than in B, because these settings may substantially differ with respect to the types of healthcare expenses, the (sub)population under analysis, the price of healthcare services, and medical technology. Therefore, in many cases we do not know whether a model with R^2^ = 0.30 reduces the predictable profits and losses more than a model with R^2^ = 0.20.

In sum, one should be careful to compare the R^2^-values across different settings and over time.

A second example is given by Van Barneveld et al. [6, p. 136–137]. They simulated on the same data set both several risk equalization models that a regulator could use and a selection model that an insurer could use. Their empirical results showed two regulator-models such that the model with the higher R^2^ resulted in higher incentives for selection (and not in lower incentives). They concluded that R^2^-values can be misleading if they are used as an indicator of an insurer’s incentives for selection.^13^

A third example is the comparison of the R^2^ of prospective and retrospective risk equalization models. *Prospective* equalization models include risk adjusters based on information that is known before the prediction period (e.g., diagnoses in the prior year), while *concurrent* equalization models use risk adjusters based on information that becomes known during the prediction period (e.g., diagnoses in the current year). The R^2^ of concurrent models is typically higher than that of prospective models because the correlation between healthcare expenses and current-year diagnoses is inherently higher than the correlation between healthcare expenses and prior-year diagnoses. However, it would be incorrect to conclude that *because of their higher R*^*2*^ concurrent models reduce the incentives for risk selection more than prospective models. Comparison of prospective and concurrent models on the basis of R^2^ alone is problematic because the R^2^-value of a concurrent model does not only capture ‘predictable’ variance in healthcare expenses but also some ‘unpredictable’ variance related to occurrence of new health problems that could not have been predicted ex-ante. So, without further information and without a relevant benchmark the difference between the R^2^ for prospective and concurrent models is hard to interpret.

These examples illustrate that the classical interpretation that a high R^2^ indicates smaller predictable profits and losses than a low R^2^, can be misleading in the context of risk equalization. Consequently, comparing alternative equalization models solely on the basis of the R^2^-value can lead to misleading conclusions about which model is to be preferred.

### Conclusion

Most empirical studies that estimate the coefficients of a risk equalization regression model (routinely) present the R^2^-value, which is often (implicitly) interpreted as a measure of the extent to which the risk equalization payments remove the regulation-induced predictable profits and losses on the insured. However, it is hard to interpret the R^2^-values as a measure of selection incentives and to compare the R^2^-values across different settings for the following reasons:

1. In nearly all studies there is no benchmark for interpreting the R^2^, because the maximum R^2^-value is unknown. Moreover, there are many determinants of the maximum R^2^, which are usually not taken into account.
2. The R^2^ is nonlinearly related to the predictable profits and losses.
3. A high R^2^ does not necessarily imply a better performance than a low R^2^. In many cases we do not know whether a model with R^2^ = 0.30 reduces the predictable profits and losses more than a model with R^2^ = 0.20.

In sum, in the context of risk equalization as a tool to reduce the predictable profits and losses the R^2^-value is hard to interpret as a measure of selection incentives, can lead to wrong and misleading conclusions when used as a measure of selection incentives,^14^ and is therefore not useful for measuring selection incentives.

## Related measures-of-fit: similar problems

Mutatis mutandis some or all the above-mentioned problems with the R^2^-values hold similarly for related statistical measures-of-fit such as the Mean Absolute Prediction Error (MAPE), Cumming’s Prediction Measure (CPM) and the Payment System Fit (PSF).

### Mean Absolute Prediction Error (MAPE)

As discussed above, the conventional R^2^ attaches enormous weight to large outliers. For example, an individual with residual spending of 1 million euros has the same impact on the R^2^-value as 1 million individuals with residual spending of 1,000 euros. A measure that does not weigh prediction errors differently, is the Mean Absolute Prediction Error (MAPE). The MAPE is calculated as the mean of the absolute value of ‘predicted expenses minus actual expenses’ across all individuals. The MAPE is less sensitive to extreme values in the distribution of expenses than the R^2^. However, it is unknown what the minimum value of the MAPE is that can be achieved with risk equalization models.

Mutatis mutandis the problems with the interpretation of R^2^-values as mentioned in the Sects. "No benchmark because the maximum R^2^-value is typically unknown" and "A high R^2^ does not necessarily imply a better performance than a low R^2^" hold similarly for the interpretation of the MAPE-value as a measure of selection incentives.

### Cumming’s prediction measure (CPM)

Another measure that does not weigh prediction errors differently, is the Cumming’s Prediction Measure (CPM), which is equal to one minus the ratio of the MAPE to the mean absolute deviation from the mean expenses [28, p.51]. Like the R^2^, its value ranges between zero and one. One could interpret the CPM as the proportion of the mean absolute deviation from the mean expenses that is explained or predicted by the linear influence of the set of risk adjusters. However, the maximum value of CPM that can be achieved with risk equalization models is unknown, because the minimum value of the MAPE is unknown.

Mutatis mutandis the problems with the interpretation of R^2^-values as mentioned in the Sects. "No benchmark because the maximum R^2^-value is typically unknown" and "A high R^2^ does not necessarily imply a better performance than a low R^2^" hold similarly for the interpretation of the CPM-value as a measure of selection incentives.

### Payment system fit (PSF)

Zhu et al. [29] introduced the measure Payment System Fit (PSF) as a generalized form of R^2^ to capture the fit of the *entire* payment system, i.e., equalization payments plus other payments, e.g., from risk sharing (i.e., ex-post cost-based payments to the insurers) and premiums. Their PSF is an R^2^-type statistical measure-of-fit that quantifies the portion of the variance in healthcare expenses that is explained by the *entire* payment system.^15^ This PSF is analogous to Efron’s R^2^ [13], which is equal to one minus the ratio of the variance of the error term to the variance of the dependent variable. Similar arguments why the R^2^ is hard to interpret as a measure of selection incentives (see above) also apply to the PSF. Although Zhu et al. [29] did not give an explicit interpretation of the calculated PSF-values when presenting their empirical results, they wrongly gave the impression that maximizing PSF (just like R^2^) is an objective of the payment policy.^16^

The PSF has also been applied in other studies, e.g., [10 and 30–34]. In contrast to Zhu et al. [29] these other studies explicitly use the PSF to quantify *selection incentives*. More specifically, these studies associate a higher PSF*-*value with reduced incentives for risk selection. However, in the case of risk sharing (as in these studies) this doesn’t have to be true (for a counterexample see Table 1). The PSF-value indicates the fit between the payments that an insurer receives and the *actual* expenses, while for an indicator of the incentives for risk selection the fit between these payments and the *predictable* expenses is relevant.

**Table 1:** A high PSF may not be preferred to a low PSF

	PSF	Reduction of the predictable profits/losses for subgroups of interest
Model 1 *	0.750	50%
Model 2 **	0.298	84%

^*^Model 1: A payment system with 50% proportional risk sharing and *without* risk equalization

^**^Model 2: A payment system with risk equalization and *without* risk sharing. This payment system mimics the risk equalization model used in the Netherlands in 2016. Source: Van Kleef et al.[20]

In general, it is unknown which portion of actual spending is predictable. Consequently, it is unknown whether and to what extent risk sharing (i.e., payments based on *actual* spending) reduces the incentives for risk selection. E.g., in the hypothetical situation that risk equalization fully compensates for predictable profits and losses, the addition of risk sharing payments can only lead to compensation of unpredictable losses and therefore does not reduce the incentives for risk selection. Nevertheless, the PSF-value (strongly) increases.^17^ The same conclusion holds in the case of a very high threshold in the risk sharing scheme such that the threshold is higher than the highest predictable individual expenses. In that case the risk sharing payments only compensate for unpredictable losses.^18^

Van Kleef et al. [20] present an example that clearly illustrates that in the case of risk sharing a higher PSF*-*value does not necessarily reduce the predictable profits and losses. A ‘payment system with 50% proportional risk sharing and *without* risk equalization’ has a PSF-value of 0.75 and reduces the predictable profits/losses for subgroups of interest by 50% (assuming that premiums are community-rated). Although systems with state-of-the-art prospective risk equalization have a PSF*-*value that is much lower (somewhere in the range of 0.30–0.35), these risk equalization models have been proven to better mitigate predictable profits and losses than 50% proportional risk sharing. For example, Van Kleef et al. [20] find that the prospective risk equalization model used in the Netherlands in 2016 has a PSF of 0.298 and reduces predictable profits/losses for subgroups of interest by 84% (see Table 1).

So, when comparing different ‘entire payment systems’ (with various degrees and forms of risk sharing) the model with the highest PSF-value does not necessarily reduce the predictable profits and losses the most, even if they are estimated on the same data.

In sum, in the context of ‘risk equalization and risk sharing as tools to reduce the predictable profits and losses’ the PSF is hard to interpret as a measure of selection incentives, is an inappropriate measure of the extent to which the payment system reduces predictable profits and losses, and is therefore not useful for measuring selection incentives.

## Applications in which R^2^ can be useful

Despite the previously discussed problems, in the following cases R^2^ can be useful.^19^

### F-test when estimating different equalization formulas on the same data

In Sect. "No benchmark because the maximum R^2^-value is typically unknown" we discussed several determinants of the (maximum) R^2^ that make it hard to compare the R^2^-values across different settings and over time. When comparing different risk equalization formulas that are defined by adding new risk adjusters to the previous formula and that are estimated by means of OLS on the *same data*, this problem does not occur. However, as discussed above, it is then still hard to interpret an increase of the R^2^-value in terms of a reduction of the selection incentives, because there is no benchmark (if, as usual, the maximum R^2^ is unknown) and because of the non-linear relation between R^2^ and the predicted profits/losses.^20^ However, the R^2^-values can be useful for calculating the F-value to perform an F-test to test whether the set of additional risk adjusters, given the set of original risk adjusters, jointly make a statistically significant contribution to further explain the variance in healthcare expenses (see e.g., [35, p. 371].

### Evaluating the validity of data used for calibration of risk equalization models

The R^2^-value can be informative at the calibration stage of risk equalization models. For example, when recalibrating a given risk equalization formula on a more recent data year, the R^2^ might be a helpful indicator to detect changes in cost patterns in the estimation data. A substantial decrease or increase in R^2^-value for the same risk equalization model when going from one data year to another might point at a change in cost structures. Researchers might want to find the source of such a change when evaluating the validity of the estimation data. For example, Cattel et al. [36] find a substantial decrease in R^2^-value of the Dutch risk equalization model when moving from data-year 2019 to data-year 2020. After further research, Cattel et al. [36] conclude that this decrease was caused by the COVID-19 pandemic. More specifically, the pandemic lead to a disproportionate increase in unpredictable spending variation. After some careful checks, however, the impact on the coefficients of the risk equalization model seemed to be limited and the researchers advised to use the 2020-data for calibration of the risk equalization model for 2023.

### Evaluating the extent to which ‘constraints’ on the estimated coefficients are binding

Risk equalization models can include constraints on the estimated coefficients. For example, the Dutch risk equalization model-2024 for somatic care includes the constraint that for a specific risk class of healthy and unhealthy people (which is not included as a risk adjuster variable) mean predicted spending equals mean actual spending.^21^ This procedure, which is known as ‘constrained regression’, generates coefficients that minimize the sum of squared residuals (given a set of risk adjusters) conditional on the constraint. When using constrained regression, the R^2^-value can be a simple, first signal for researchers that indicates the extent to which a restriction is binding, i.e., the extent to which the constraint requires deviations from the original OLS-estimates. If the constraint is not binding (because it is already fulfilled in the original OLS without a constraint), the R^2^ remains the same. The more a constraint is not fulfilled with the original-OLS-estimated coefficients, the more will the constrained-OLS-estimated coefficients of the risk adjusters that are correlated with the constraint-factor, (have to) deviate from the original-OLS-estimated coefficients, and therefore the lower will be the R^2^ of the constrained regression. In that case, researchers may take a close look at other measures, e.g., the (change in) over/undercompensations of risk groups that are used as risk adjusters. In the case of OLS there are no over/undercompensations of these risk groups. However, in the case of constrained regression this is no longer the case, and the over/undercompensations for these groups create new incentives for risk selection, which may not be acceptable for the regulator.

### Economic rather than statistical measures-of-fit

The statistical R^2^ measure does not give any information about (1) whether in practice selection will occur, (2) and if so, which forms of selection, and (3) what negative effects selection will have. Therefore, in addition to the *statistical* R^2^ measure Layton et al. [21] developed an *economic* R^2^-type measure to indicate the expected welfare effects of risk selection. In doing so, they made assumptions about how financial incentives influence the behavior of insurers and consumers, how this can result in price and benefits distortions, and how these distortions result in welfare loss. Although this economic R^2^-type measure is useful, in our opinion it should always be carefully described and interpreted in the light of the underlying economic model and assumptions (to avoid misinterpretations by readers who are not yet familiar with that measure).

## Conclusion and discussion

Nearly all empirical studies that estimate the coefficients of a risk equalization formula present the value of the statistical measure R^2^, mostly without a clear interpretation. Often the R^2^-value is implicitly interpreted as a measure of the extent to which the risk equalization payments remove the regulation-induced *predictable* profits and losses on the insured, i.e., a measure of selection incentives.

In this paper we have argued that in the context of risk equalization R^2^ is hard to interpret as a measure of selection incentives, can lead to wrong and misleading conclusions when used as a measure of selection incentives, and is therefore not useful for measuring selection incentives. In many cases, we do not know whether a model with R^2^ = 0.30 reduces the predictable profits and losses more than a model with R^2^ = 0.20. So, although in the context of risk equalization research the R^2^ is the most used measure-of-fit (Van Veen et al., 2015) and is often implicitly interpreted as a measure of selection incentives, it is not useful for measuring selection incentives. Similar problems hold for related statistical measures-of-fit such as the MAPE, CPM, and the PSF. There are some exceptions where the R^2^ can be useful.

Our conclusion is that one should be careful with presenting and interpreting R^2^-values in risk equalization research.

### How to evaluate the performance of risk equalization models?

Given our conclusion that the R^2^ is not useful for measuring selection incentives, one could raise the question: Which evaluation measure can be used to appropriately evaluate the performance of risk equalization models?

Van Veen et al. [12] and Layton et al. [24] give an overview of statistical measures for evaluating the predictive performance of risk equalization models. Van Veen et al. [12] give an overview of statistical measures-of-fit that have been used for evaluating risk equalization models since 2000 and discussed the properties of 71 measures-of-fit. They concluded that the R^2^ is the most commonly used measure. This underlines the relevance of our paper. Van Veen et al. [12] conclude that “if the objective is measuring financial incentives for risk selection, the only adequate evaluation method is to assess the performance for selected nonrandom groups of interest” (different from the groups formed by the risk adjusters). If the groups are sufficiently large, the difference between the average equalization-based predicted expenses and the average actual expenses for relevant non-random groups (e.g., the chronically ill or healthy people) can be interpreted as the over/undercompensations (i.e., predictable profit or loss) for an individual in that group. Therefore, a good evaluation measure of the performance of risk equalization models is the over/undercompensations for relevant non-random groups.

Alternatively, predictive ratios for relevant non-random groups are presented in the literature [24]. A predictive ratio equals the ratio of the average equalization-based predicted expenses to the average actual expenses for a relevant non-random group. For the following reason we prefer the over/undercompensations over the predictive ratios to assess the performance of a risk equalization model for selected non-random groups. A predictive ratio is hard to interpret as a single measure of selection incentives because the selection incentive (i.e., the over/undercompensation per group) depends on both the predictive ratio and the average actual expenses of the group. So, one should also know the latter to calculate a measure of selection incentives. One could hold the view that when comparing the predictive ratios across groups a predictive ratio closer to 1 is associated with a smaller selection incentive. However, this is not necessarily correct, as is illustrated by the example in Table 2.

**Table 2:** A Predictive Ratio closer to 1 does not necessarily imply a smaller selection incentive

Group	Predictive ratio	Average actual expenses (in euro)	Selection incentive = average predictable profit = (predictive ratio—1) * average actual expenses
A	1.1	4000	400
B	1.2	4000	800
C	1.3	1000	300

Because there are many different relevant non-random groups, the question is for which non-random groups should a regulator calculate the over/undercompensations. The answer depends on the regulators’ objective of the payment system. Regulators may give more priority to preventing ‘quality skimping’ via the distortion of healthcare services by the insurers than to preventing selective marketing. Therefore, a regulator may give a relatively high weight to the undercompensation of groups that are vulnerable to quality skimping (e.g., chronically ill people). An option is to calculate a weighted average of the absolute over/undercompensations of a set of relevant nonrandom groups as a single measure of the performance of a risk equalization model (see e.g., [20, 38]). For a comprehensive framework for an ex-ante evaluation of the (positive and negative) effects of risk equalization see [27].

### Why maximizing R^2^?

Given our conclusions about R^2^, one could raise the question: Does it make sense, as we usually do, to maximize the R^2^-value (i.e., using the OLS regression method) when estimating the coefficients of a risk equalization formula? Our answer is: yes. It is important to make a distinction between the role of R^2^ in the *estimation method* and the R^2^ as an *evaluation measure* of the performance of a risk equalization model. Our criticism of the R^2^ relates only to the R^2^ as an evaluation measure of the selection incentives.

*If* we specify a *linear* relation between individual health expenses and the set of risk adjusters, it turns out that under the classical assumptions^22^ both the Best Linear Unbiased (BLU) estimators and the Maximum Likelihood (ML) estimators are equivalent to the OLS estimators of the regression parameters (see e.g., [35, p. 205–216]. This implies that under the classical assumptions the OLS estimators are unbiased, have the smallest variance among all linear unbiased estimators, are consistent and asymptotically efficient.

Therefore, it is important to emphasize that *maximizing the R*^*2*^* is not an objective of the payment policy or an end in itself; it is just a means to get estimators with very desirable statistical properties (BLU and ML).*^23^ An objective of the payment policy is to eliminate the regulation-induced incentives for risk selection by compensating insurers for *predictable* profits and losses [2]. There are many ways to estimate the coefficients for predicting the individual health expenses (which is necessary for compensating insurers for predictable profits and losses) and in practice OLS, i.e. maximizing the R^2^, is the mostly used method to do so. However, the fact that the R^2^ is maximized to be able to calculate predicted expenses, does not make the R^2^ an appropriate measure of selection incentives.

Another question could be: *Why* do we specify a *linear* relation between individual health expenses and the set of risk adjusters? There are two good arguments to do so. First, by specifying a linear relation we stay close to the cell-based approach. Assume that there is only one risk adjuster, e.g., age with, say, 24 classes. Then most regulators would use the cell-based approach, i.e., use the average expenses per age group as the basis for calculating the equalization payments (as in e.g., Colombia [40], Israel [41], and in Switzerland till 2020 [42]). If in addition to age a second risk adjuster is relevant, e.g., yes/no healthy, most regulators would use the cell-based approach with 24 × 2 = 48 cells. The results of these cell-based approaches are exactly the same as the results of OLS with these 24 respectively 48 binary (0/1) risk adjusters as dependent variables and no intercept. When there are many additional risk adjusters the number of cells may soon become too large. A problem is then that in many cells there are too few observations to get reliable results. In addition, many coefficients may not be stable over time. A solution is to make the assumption (which is implicitly made in nearly all risk equalization models in practice) that most or all interaction terms between the risk adjusters are zero. For example, the assumption can be made that the effect of health on expenses is the same for each age group. The weights of the equalization formula can then be estimated by applying OLS to a linear relation between individual expenses and a manageable number of binary (0/1) risk adjusters.

A second argument to specify a *linear* relation between individual health expenses and the set of risk adjusters is that a linear relation avoids the complicated retransformation problems with making predictions of the individual health expenses when using a *nonlinear* relation [43]. Examples of nonlinear models include two-part models^24^ or nonlinear transformations of the health expenses such as the logarithm of health expenses (see e.g., [44]). However, with the two-part models the predicted expenses are seriously biased in the case of heteroscedasticity, and the use of the simple transformation log(expenses + 1) has very poor statistical properties when calculating the predicted individual expenses by retransforming to the original scale (e.g., euros rather than log-euros) [45, 46]. According to Mullahy [46] applying OLS to individual health expenses may be sufficient when sample sizes are large.

### A high R^2^ is not necessarily preferred to a low R^2^

In Sect. "A high R^2^ does not necessarily imply a better performance than a low R^2^" we concluded that a high R^2^ does not necessarily imply a better performance than a low R^2^. However, there are also other reasons why a high R^2^ is not necessarily preferred to a low R^2^.

A first example is constrained regression. The goal of risk equalization is to eliminate the regulation-induced incentives for risk selection by compensating insurers for predictable profits and losses [2]. However, as long as the risk equalization is imperfect, the regulator may analyze the potential effects of different selection incentives (that result from the over/undercompensations for relevant non-random groups) on the insurers’ and consumers’ behavior, analyze what types of selection actions are possible and realistic, and what the negative effects of these selection actions are. For example, regulators might be particularly concerned about risk selection via the distortion of insurance products and via quality skimping [47]. The regulator could then give priority to reduce the predictable profits and losses that induce the selection actions with the most negative effects. One tool to do so is constrained regression, which, by definition, results in a lower R^2^ than OLS. For example, Van Kleef et al. [37] present results showing that adding specific constraints to an OLS-regression model reduces the R^2^, but strongly reduces the predictable losses for subgroups of chronically ill people and thereby reduces the incentives for quality skimping.^25^ The regulator may prefer the model with a worse *statistical* performance (in terms of R^2^) but better *economic* performance (i.e., fewer selection incentives regarding groups of interest) to the model with better *statistical* performance but worse *economic* performance. That is, the regulator may prefer the model-with-low-R^2^ to the model-with-high-R^2^.

A second example is optimal risk adjustment. In the literature two different approaches for estimating the weights of a risk equalization model can be discerned given a set of risk adjusters and risk classes (that result in imperfect risk equalization): the so-called ‘conventional risk adjustment’ and ‘optimal risk adjustment’. Layton et al. [48] describe *conventional* risk adjustment as a *two-step* “estimate-then-evaluate” approach: first the weights for a given risk equalization model are estimated and then second the risk equalization model is evaluated based on certain criteria that reflect the regulator’s objective. *Optimal* risk adjustment is described as a *one-step* “estimate-to-maximize-the-objective” approach, where the payment weights are estimated such that the regulator’s objective function is maximized.^26^ If the R^2^ for optimal risk adjustment is lower than the R^2^ for conventional risk adjustment with the weights estimated by OLS,^27^ the regulator will prefer the low R^2^ to the high R^2^.

### Why do we use R^2^ so often?

Because most of the problems with the R^2^ (as mentioned in this paper) have been known for decades, the question comes to mind: *Why do we use R*^*2*^* so often in risk equalization research?* Unfortunately, for most studies^28^ we do not know the answer to this question. A possible answer could be that there is no other performance measure available. In that case it is recommended to invest in the collection of appropriate data to calculate useful statistical measures such as the average over/undercompensations per relevant selected non-random group (e.g., chronically ill or healthy people)” [12].

In general, our recommendation is to either present the R^2^ with a clear, valid, and relevant interpretation or not to present the R^2^. The same holds for the related statistical measures MAPE, CPM, and PSF.

## Data Availability

There are no data sets used for this article.

## Footnotes

1 *Selection* can be defined as “actions by consumers and insurers to exploit unpriced risk heterogeneity and break pooling arrangements” [1]. The insurers’ incentives for risk selection are determined by both the regulation-induced *predictable* profits and losses on the insured and the insurer’s costs of selection, e.g., a reduction of good reputation and the costs of the selection activities (e.g., finding out who are the (un)profitable people and designing, marketing, and administering new insurance products to deter unprofitable people and attract profitable people). In this paper we will abstract from these costs and only focus on predictable profits and losses.

2 For reasons of fairness there may be some exceptions, e.g., if predictable profits reflect underutilization [3, p. 783–784], [4].

3 Achieving a level playing field for insurers and achieving maximum cross-subsidies among consumers require that all risk factors that insurers are not allowed to use for premium differentiation should be included in the risk equalization [2].

4 The R^2^ can be calculated as: the variance of predicted expenses divided by the variance of actual expenses, or the square of the correlation between predicted expenses and actual expenses, or one minus the ratio of the variance of the prediction error divided by the variance of actual expenses (where the prediction error equals the difference between predicted expenses by the risk equalization model and actual expenses) [12]. These three ways of calculating the R^2^ are only equivalent when an OLS-model is used and expenses are predicted on the full sample. If this is not the case, the only correct way of calculating the R^2^ is the third method (i.e., the R^2^ proposed by Efron [13]).

5 Ideally, to prevent overfitting R^2^-values should be reported which are based on out-of-sample predictions. In that case Efron’s [13] R^2^ should be used. The out of sample R^2^ is not bounded by zero.

6 This paper is partly based on several previous papers by the authors and their colleagues (see the references). The novelty of this paper is that it brings together some relevant R^2^-related aspects of these previous papers, builds upon them, extends the analysis, provides several new arguments and insights, and comes with new conclusions and recommendations concerning the R^2^ in the context of risk equalization.

7 The R^2^ of *prospective* risk equalization models captures only predictable variance, in contrast to the R^2^ of *concurrent* risk equalization models that also captures unpredictable variance. To our knowledge there is no study on the maximum R^2^ for concurrent models.

8 Other studies have analyzed the extent of predictable profits and losses that remain after risk equalization by simulating a ‘best available’ prediction model for insurers. For different models that the regulator could use, these studies present the predictable profits and losses that insurers could make with their ‘best available’ prediction model. Although these studies are informative, they underestimate the potential profits (losses) that insurers could make (avoid) because in practice insurers and consumer most likely have more predictive information than is included in these ‘best available’ models. Examples of such studies are [5–9].

9 For a nontechnical description of this method see [15].

10 For two of the rare examples of studies in which the maximum R^2^ has been calculated, see [6, p. 426] and [7,p. 134].

11 In these studies by Van Barneveld et al. [7] and Newhouse et al. [14] the maximum R^2^ was estimated.

12 Alternatively, one can apply measures such as the Mean Absolute Prediction Error (MAPE) and Cumming’s Prediction Measure (CPM), which both are a function of absolute deviations, rather than squared deviations (see Sect. "Mean Absolute Prediction Error (MAPE)" and "Cumming’s Prediction Measure (CPM)").

13 For their empirical analyses Van Barneveld et al. [6] used a sample of 47,000 individuals. They did not present confidence intervals of the insurer’s financial results that were interpreted as incentives for selection. So, it is unknown whether their conclusion is based on statistically significant results.

14 The following example is another illustration how the presentation of the R^2^-value can lead to wrong and misleading conclusions. The Dutch government repeatedly tried to convince the Parliament that the risk equalization works well by showing that the R^2^ in analyses explaining the “cost variation *among insurers’ portfolios*” is 0.98 [25, 26]. However, this is an incorrect argument. A problem with the R^2^ and other measures based on the variation in (residual) spending at the *insurer level* is that the outcomes of these measures heavily depend on the distribution of risk types across insurers’ portfolios and on the cost structure in these portfolios in the specific data set used for evaluation [27]. For example, consider the worst risk equalization formula with for each person the predicted expenses equal to the mean per person expenses. Such a risk equalization model has maximum predictable profits and losses. Nevertheless, if the risk composition and cost structure are identical across the insurers’ portfolios, the R^2^ at the *insurer level* is 1.0. So, the R^2^ at the *insurer level* is misleading because it is not a valid measure of predictable profits and losses on the insured.

15 To our knowledge Zhu et al. [29] were the first to introduce this Payment System Fit (PSF).

16 Zhu et al. [29, p. 216] motivated the introduction of the Payment System Fit (PSF) as follows: “One objective of payment policy is to match payments to expected costs for individuals. Risk adjustment systems are commonly graded by their R-squared, a statistic reporting how much of the variation in health care costs is explained by the variables in the regression underlying the risk adjustment formula. Our generalization of the statistical R-Squared metric reflects how much of the total variation in plan-paid costs is captured by all payment-system features.” They rightly stated that an objective of payment policy is to match payments to *expected* costs for individuals. However, as argued above, the R^2^ measures the fit between payments and *actual* costs, and the same holds for the PSF. Therefore, Zhu et al. [29] wrongly gave the impression that maximizing PSF (just like R^2^) is an objective of the payment policy.

17 In fact, in the case of perfect risk equalization risk sharing might *increase* the incentives for selection depending on the financing of the risk sharing payments. For different forms of risk sharing and different forms of financing the risk sharing payments see e.g., [3].

18 Most likely, in practice we do not know what the ‘highest predictable individual expenses’ are.

19 Mutatis mutandis the same holds for MAPE and CPM; not for PSF.

20 As discussed in Sect. "R2 is nonlinearly related to the predictable profits and losses" this non-linear relation can lead to misleading conclusions in the case of *small* predictable profits and losses.

21 This type of restriction has been tested in several papers, see e.g., [37–39].

22 For each individual the error is normally distributed, with expectation zero and variance sigma (homoskedasticity), and is not correlated with the error terms of other individuals (see e.g., [35]).

23 Even if the classical assumptions (see previous footnote) are not fulfilled, the OLS-estimators have desirable properties. If the error is not normally distributed, the OLS-estimators are still the BLU estimator, i.e., they are unbiased and have the smallest variance among all linear unbiased estimators. In the case of heteroscedasticity, the OLS estimators are still unbiased and asymptotically efficient (see e.g., [35, Chapter 8].

24 In the first part, a yes–no expenses equation is estimated (e.g., a probit, logit, or linear probability model). In the second part, for those with positive expenses, the expenses are regressed (a linear, log-linear or square root model) on the risk adjusters (see e.g., [44–46]). Conventionally, both parts of the two-part model are estimated independently and a smearing transformation (see [43]) is used to generate unbiased estimates of the second part of the model in the common situation in which nonlinear transformations of the dependent variable are used.

25 This example shows that even when comparing different risk equalization formulas that are estimated on the same data, it is hard to interpret the R^2^ without further relevant information, because a low R^2^ may be preferred to a high R^2^.

26 If the regulator’s objective is perfectly specified, maximizing this objective function under realistic assumptions would be sufficient and would make a further evaluation superfluous. However, several potential objectives of the regulator can be discerned, which in ‘optimal risk adjustment’ (so far) are maximized without considering the other objectives. Glazer and McGuire [49] and Layton et al. [48] develop a framework where the objective is to minimize the efficiency loss from service-level distortions due to adverse selection (i.e., a form of *indirect* selection). This objective is relevant for countries such as the United States of America and the Netherlands, where *individual* insurers purchase or deliver the care to their own enrollees, but this objective seems less relevant for countries such as Germany and Switzerland, where the insurers *collectively* purchase the care. Lorenz [50] analyzes optimal risk adjustment where the objective is to eliminate *direct* selection (e.g., selective advertising, or avoiding people living in high-cost areas). In practice, however, regulators might also be concerned with other objectives, such as a level playing field for insurers, the desired level of cross-subsidies, no adverse selection into/out the market by consumers, avoiding other negative effects of risk selection, and basing equalization payments only on acceptable costs (see e.g., [3]). According to Layton et al. [48] a formal incorporation of *all* regulator’s objectives within a single social welfare function is probably unrealistic. Therefore, also in the case of optimal risk adjustment, after the *first* step of estimating the payment weights, a *second* step is desirable to evaluate the performance of the risk equalization model.

27 As is the case in the empirical analyses by Glazer and McGuire [49], Lorenz [50] and Layton et al. [48].

28 In Sect. "Applications in which R2 can be useful" we discussed some applications in which R^2^ can be useful. However, most studies on risk equalization that present the R^2^ fall outside the scope of these applications.

## References

[1] Newhouse, J.P.: Reimbursing health plans and health providers: efficiency in production versus selection. J. Econ. Literature **34**, 1236–1263 (1996)

[2] Van de Ven, W.P.M.M., Hamstra, G., van Kleef, R., Reuser, M., Stam, P.: The goal of risk equalization in regulated competitive health insurance markets. Eur. J. Health Econ. **24**, 111–123 (2023) 35348921 10.1007/s10198-022-01457-7 PMC9877069

[3] Van de Ven, W.P.M.M. & R.P. Ellis. (2000). Risk adjustment in competitive health insurance markets. In: Culyer AJ, Newhouse JP, editors. *Handbook of Health Economics* (Chapter 14) Amsterdam: Elsevier, 755–845.

[4] McWilliams, J.M., Weinreb, G., Ding, L., Ndumele, C.D., Wallace, J.: Risk adjustment and promoting health equity in population-based payment: concepts and evidence. Health Aff. **42**(1), 105–114 (2023) 10.1377/hlthaff.2022.00916 PMC9901844 36623215

[5] Lamers, L.M.: Risk-adjusted capitation based on the diagnostic cost group model: an empirical evaluation with health survey information. Health Serv. Res. **33**(6), 1717–1728 (1999) PMC1070345 10029506

[6] Lamers, L.M.: Health-based Risk adjustment: is inpatient and outpatient diagnostic information sufficient? Inquiry **38**(4), 423–431 (2001) 11887959 10.5034/inquiryjrnl_38.4.423

[7] Van Barneveld, E.M., Lamers, L.M., van Vliet, R.C.J.A., van de Ven, W.P.M.M.: Ignoring small predictable profits and losses: a new approach for measuring incentives for cream skimming. Health Care Manag. Sci. **3**, 131–140 (2000) 10780281 10.1023/a:1019029004807

[8] Van Barneveld, E.M., Lamers, L.M., van Vliet, R.C.J.A., van de Ven, W.P.M.M.: Risk sharing as a supplement to imperfect capitation: a tradeoff between selection and efficiency. J. Health Econ. **20**(2), 147–168 (2001) 11252368 10.1016/s0167-6296(00)00077-1

[9] Shen, Y., Ellis, R.P.: How profitable is risk selection? A comparison of four risk adjustment models. Health Econ. **11**, 165–174 (2002) 11921314 10.1002/hec.661

[10] Van Kleef, R.C., van Vliet, R.C.J.A.: How to deal with persistently low/high spenders in health plan payment systems? Health Econ. **31**, 784–805 (2022) 35137476 10.1002/hec.4477 PMC9305280

[11] Van Kleef, R.C., R.C.J.A. van Vliet, M. Oskam. (2024) Risk Adjustment in Health Insurance Markets: Do Not Overlook the “Real” Healthy. *Medical Care* forthcoming. 10.1097/MLR.0000000000001955 PMC11462869 38047754

[12] Van Veen, S.C.H.M., van Kleef, R.C., van de Ven, W.P.M.M., van Vliet, R.C.J.A.: Is There one measure-of-fit that fits all? A taxonomy and review of measures-of-fit for risk-equalization models. Med. Care Res. Rev. **72**, 220–243 (2015) 25694164 10.1177/1077558715572900

[13] Efron, B.: Regression and ANOVA with zero-one data: measures of residual variation. J. Am. Stat. Assoc. **73**, 113–121 (1978)

[14] Newhouse, J.P., Manning, W.G., Keeler, E.B., Sloss, E.M.: Adjusting capitation rates using objective health measurers and prior utilization. Health Care Financ. Rev. **10–3**, 41–54 (1989) PMC4192960 10313096

[15] Newhouse, J.P., Buntin, M.B., Chapman, J.D.: Risk adjustment and medicare: taking a closer look. Health Aff. **16**(5), 26–43 (1997) 10.1377/hlthaff.16.5.26 9314674

[16] Van Vliet, R.C.J.A.: Predictability of individual health care expenditures. J. Risk Insur. **59**(3), 443–460 (1992)

[17] Kronick, R., Zhou, Z., Dreyfus, T.: Making risk adjustment work for everyone. Inquiry **32**, 41–55 (1995) 7713617

[18] Newhouse, J.P., Sloss, E.M., Manning, W.G., Keeler, E.B.: (1993) Risk adjustment for a children’s capitation rate. Health Care Financing Review, Fall **15**(1), 39–54 (1993) PMC4193411 10133708

[19] Van Kleef, R.C., Eijkenaar, F., van Vliet, R.C.J.A., van de Ven, W.P.M.M.: Health plan payment in the Netherlands. In: McGuire, T.G., van Kleef, R.C. (eds.) Risk Adjustment, Risk Sharing and Premium Regulation in Health Insurance Markets, Theory and Practice (Chapter 14) Academic Press, pp. 397–429. Elsevier, London (2018)

[20] Van Kleef, R.C., Eijkenaar, F., van Vliet, R.C.J.A.: Selection incentives for health insurers in the presence of sophisticated risk adjustment. Med. Care Res. Rev. **77**, 584–595 (2020) 30704337 10.1177/1077558719825982 PMC7536529

[21] Layton, T.J., Ellis, R.P., McGuire, T.G., van Kleef, R.C.: Measuring efficiency of health plan payment systems in managed competition health insurance markets. J. Health Econ. **56**, 237–255 (2017) 29248054 10.1016/j.jhealeco.2017.05.004 PMC5737816

[22] Harberger, A.C., (1964), Principles of efficiency; the measurement of waste *American Economic Review* 54(3): 58–76.

[23] Van Kleef, R.C. (2022). *Socioeconomic Variables in Risk Adjustment Experiences from the Netherlands.* Presentation at the RAN-Meeting, Berlin, September 2022.

[24] Layton, T.J., R.P. Ellis, T.G. McGuire, R.C. van Kleef. (2018) Evaluating the Performance of Health Plan Payment Systems. Chapter 5 in T.G. McGuire and R.C. van Kleef *Risk adjustment, risk sharing, and premium regulation in health insurance markets, Theory and Practice*. Elsevier, Academic Press, 133–167.

[25] Tweede Kamer (2011a) Herziening zorgstelsel. Brief van de minister aan de Tweede Kamer, 29689(350).

[26] Tweede Kamer (2011b) Herziening zorgstelsel. Verslag, 29689(358).

[27] Van Kleef, R.C., Reuser, M., Stam, P.J.A., van de Ven, W.P.M.M.: A framework for ex-ante evaluation of the potential effects of risk equalization and risk sharing in health insurance markets with regulated competition. Health Econ. Rev. **14**(1), 57 (2024) 39046547 10.1186/s13561-024-00540-4 PMC11267970

[28] Cumming, R.B., D. Knutson, B.A. Cameron, and B. Derrick. (2002) *A Comparative Analysis of Claims Based Risk Assessment for Commercial Populations,* Society of Actuaries, USA. https://www.researchgate.net/publication/228378875_A_Comparative_Analysis_of_Claims_Based_Risk_Assessment_for_Commercial_Populations

[29] Zhu, J.M., Layton, T., Sinaiko, A.D., McGuire, T.G.: The power of reinsurance in health insurance exchanges to improve the fit of the payment system and reduce incentives for adverse selection. Inquiry **50**(4), 255–274 (2013) 24996751 10.1177/0046958014538913

[30] Geruso, M., McGuire, T.G.: Tradeoffs in the design of health plan payment systems: fit, power and balance. J. Health Econ. **47**, 1–19 (2016) 26922122 10.1016/j.jhealeco.2016.01.007 PMC4836985

[31] Beck, K., Kauer, L., McGuire, T.G., Schmid, C.P.R.: Improving risk-equalization in Switzerland: effects of alternative reform proposals on reallocating public subsidies for hospitals. Health Policy **124**, 1363–1367 (2020) 33008656 10.1016/j.healthpol.2020.08.011

[32] McGuire, T.G., Schillo, S., van Kleef, R.C.: Very high and low residual spenders in private health insurance markets: Germany, The Netherlands and the U.S. Marketplaces. Eur. J. Health Econ. **22**, 35–50 (2021) 32862358 10.1007/s10198-020-01227-3 PMC7822791

[33] Schmid, C.P.R., Beck, K.: Re-insurance in the Swiss health insurance market: fit, power and balance. Health Policy **120**, 848–855 (2016) 27157115 10.1016/j.healthpol.2016.04.016

[34] Henriquez, J., R.C. van Kleef, A. Matthews, T. G. McGuire, F Paolucci (2023) *Combining risk adjustment with risk sharing in health plan payment systems: private health insurance in Australia.* NBER Working paper. 10.1016/j.healthpol.2026.105584 41724136

[35] Kmenta, J.: Elements of Econometrics. Macmillan Publisher Co, New York (1971)

[36] Cattel, D., F. Eijkenaar, M. Oskam, A. Panturu, R.C. van Kleef & R.C.J.A. van Vliet. (2022). *Onderzoek Risicoverevening 2023: Overall toets.* (Research for the risk equalization model 2023: calibrating the model on the new data year.) Erasmus University Rotterdam. Research report.

[37] Van Kleef, R.C., McGuire, T.G., van Vliet, R.C.J.A., van de Ven, W.P.M.M.: Improving risk equalization with constrained regression. Eur. J. Health Econ. **18**, 1137–1156 (2017) 27942966 10.1007/s10198-016-0859-1 PMC5641290

[38] Withagen-Koster, A.A., van Kleef, R.C., Eijkenaar, F.: Incorporating self-reported health measures in risk equalization through constrained regression. Eur. J. Health Econ. **21**, 513–528 (2020) 31916028 10.1007/s10198-019-01146-y PMC7214515

[39] McGuire, T.G., Zink, A.L., Rose, S.: Improving the performance of risk adjustment systems: constrained regressions, reinsurance, and variable selection. Am. J. Health Econ. **7**, 497–521 (2021) 34869790 10.1086/716199 PMC8635414

[40] Bauhoff, S.,I. Rodriguez-Bernate, D. Gopffarth, R.Guerrero, I. Galindo-Henriquez and F. Nates. (2018) Health plan payment in Colombia. Chapter 10 in T.G. McGuire and R.C. van Kleef *Risk adjustment, risk sharing, and premium regulation in health insurance markets, Theory and Practice*. Elsevier, Academic Press, 279–294.

[41] Brammli-Greenberg, S., J. Glazer, A. Shmueli. (2018) Regulated competition and health plan payment under the National Health Insurance Law in Israel – The unfinished story. Chapter 13 in T.G. McGuire and R.C. van Kleef *Risk adjustment, risk sharing, and premium regulation in health insurance markets, Theory and Practice*. Elsevier, Academic Press, 365–395.

[42] Schmid, C.P.R., K. Beck and L. Kauer. (2018) Health plan payment in Switzerland. Chapter 16 in T.G. McGuire and R.C. van Kleef Risk adjustment, risk sharing, and premium regulation in health insurance markets, Theory and Practice. Elsevier, Academic Press, 453–489.

[43] Duan, N., et al.: Smearing estimate: a nonparametric retransformation method. J. Am. Stat. Assoc. **78**, 605–690 (1983)

[44] Manning, W.G., et al.: Health Insurance and the demand for medical care: evidence from a randomized experiment. Am. Econ. Rev. **77**, 251–277 (1987) 10284091

[45] Manning, W.G.: The logged dependent variable, heteroskedasticity, and the retransformation problem. J. Health Econ. **17**(3), 283–296 (1998) 10180919 10.1016/s0167-6296(98)00025-3

[46] Mullahy, J.: Much ado about two: reconsidering retransformation and the two-part model in health econometrics. J. Health Econ. **17**(3), 247–282 (1998) 10180918 10.1016/s0167-6296(98)00030-7

[47] Van de Ven, W.P.M.M., van Kleef, R.C., van Vliet, R.C.J.A.: Risk selection threatens quality of care for certain patients; lessons from europe? Health insurance exchanges. Health Aff. **34**, 1713–1720 (2015) 10.1377/hlthaff.2014.1456 26438748

[48] Layton, T.J., McGuire, T.G., van Kleef, R.C.: Deriving risk adjustment payment weights to maximize efficiency of health insurance markets. J. Health Econ. **61**, 93–110 (2018) 30099218 10.1016/j.jhealeco.2018.07.001 PMC6471663

[49] Glazer, J., McGuire, T.G.: Setting health plan premiums to ensure efficient quality in health care: minimum variance optimal risk adjustment. J. Public Econ. **84**, 153–173 (2002)

[50] Lorenz, N.: Using quantile and asymmetric least squares regression for optimal risk adjustment. Health Econ. **26**, 724–742 (2017) 27291046 10.1002/hec.3352

